# Our New York Letter

**Published:** 1891-04

**Authors:** Wm. L. Russell

**Affiliations:** New York


### OUR NEW YORK LETTER.

NEW YORK, March 15, 1891.

During the past two years important changes have been made in the lunacy laws of this State, which cannot fail to interest medical men throughout the world, as it is said that these laws are now among the best ever enacted. It is impossible to do more than refer briefly to the main features of the changes mentioned, and to suggest to those especially interested in the subject that a further study would be found exceedingly profitable. Two years ago the legislature created a State Commission in Lunacy, provided for the extension of the State Hospital accommodations, and changed the corporate titles of the State institutions for the insane to that of State Hospital, thus recognizing and establishing the hospital idea. This was done in accordance with the principle assumed in this State a number of years ago, that all insane persons were to be regarded as under the special care and protection of the State. The Commission in Lunacy consists of three members. The medical member, who is the chairman, the law requires to be a physician who has been in active practice for ten years and who has had some experience in the management of a hospital for the insane; the second member is a lawyer, and the third a layman, presumably a business man. They are salaried officers paid by the State, and are allowed wide scope in all matters pertaining to the care of the insane. The Commission has been in existence about twenty months, and has already done much good work. An important part of it consisted in an inspection of the county institution for the care of the insane poor. Their report revealed such a scandalous condition of affiairs, that the Legislature pursued the principle of State care of the insane still further in the passage of an act providing that all insane persons who were a public charge should be confined only in institutions supported by the State.

Thus all the insane in the State whether in public or private institutions are now under the supervision of the Commission in Lunacy, who have full authority to secure the best conditions for the safety and comfort of the patients, and the security and satisfaction of the public.

THE STUDY OF INEBRIETY.

A series of interesting meetings have been held here this winter by the newly organized Association for the Study of Inebriety. At the last meeting the question of etiology was considered. A paper was read by Dr. L. D. Mason, of Brooklyn. He believed that heredity was the most potent predisposing cause, maternal inebriety having the most decided influence. This might produce miscarriage, or the offspring might be idiots or imbeciles or the victims of some neurosis. The moderate drinker whose tissues were bathed every day in alcohol, although never getting drunk, might have just as baleful an influence on the offspring. Acquired inebriety should be separated entirely from the hereditary. Head injuries, neurasthenia, any condition producing unrest and insomnia, might be regarded as exciting causes. Alcohol was certainly a useful hypnotic in the insomnia produced by grief or worry, but it was decidedly dangerous. Dr. J. B. Mattison followed with a paper on narcotic inebriety from the use of opium, chloral or cocaine. He believed that most cases of this variety of inebriety were produced first by the prescriptions of physicians for the drugs referred to, principally in painful affections and insomnia. A demand was thus created which led the way to the habit. When cocaine was introduced it was claimed that a cure for opium addiction had been found. Many victims were led to employ it with this hope, but only to find themselves slaves of a new and worse master. In his experience the cocaine habit was much worse than that of opium or chloral. In discussing Dr. Mattison's paper, Dr. Mason agreed with the former regarding the danger arising from careless prescribing or continuance of narcotics, and he believed that the druggists were responsible for many cases from indiscriminate renewing of prescriptions which ought not to be repeated except on direct orders from the physicians. He also denounced the many patent medicines which found such a ready sale, many of which were of a very dangerous character. He said that the association had tried unsuccessfully to have a law introduced compelling the manufacturers to print the formula on each bottle. Most, if not all the so-called "cures" for the opium and alcohol habits consisted of opium or alcohol.

DIPHTHERIA.

At the last meeting of the Academy Section on Pædiatrics, Prof. J. Lewis Smith read a paper on the contributions of 1890 to our knowledge of diphtheria. He stated that he had derived the material for his paper from several volumes of extracts from periodicals, which had been placed in his hands for the purpose of preparing the article on diphtheria for the next volume of *Sajous' Medical Annual.* He would confine himself on this occasion largely to the important question of etiology. It had been discovered through researches in the Pasteur Institute that the Klebs-Lefler bacillus, which was now regarded as the true micro-organism of diphtheria, secreted ptomaines which were the direct cause of the systemic infection resulting in the well known blood changes, the nephritis and the paralysis. It was believed that other micro-organisms were capable of producing a membranous or pseudo-diphtheritic exudation, but with this systemic infection did not occur. Such an exudation sometimes appeared in the early stages of scarlet fever. It showed no tendency to invade the larynx, and did not communicate diphtheria to other children. Occasionally, however, true diphtheria supervened as the scarlet fever was declining.

Some interesting observations had been made by Klein, regarding the manner in which diphtheria might be conveyed. He had noticed that cats were sometimes affected with a bronchopneumonia, which in some instances was followed by paralysis of the hind legs. On *post mortem* examination large white kidneys were discovered due to fatty degeneration. This disease of cats produced diphtheria in children. Cultures of the Klebs-Lefler bacillus were introduced into the trachea of cats, and subsequently a *post mortem* examination revealed a membrane lining the bronchi and air-cells, and the large white kidneys before referred to. One interesting incident was related, in which a boy sick with diphtheria had vomited, and the family cat had licked at the vomited matter. The cat soon afterwards developed symptoms which were thought to resemble those of the boy. It was immediately killed, but not before it had mingled with a number of cats belonging to neighbors. Several of the cats subsequently became sick, and a child of a neighbor to whom one of them belonged developed diphtheria and died. It was known also that pigeons, turkeys and common barnyard fowls were subject to diphtheria, and communicated it to human beings.

Some observations had been made regarding the vitality of the bacillus. It had been found that a culture preserved for sixteen months had lost none of its virulence. There were several incidents illustrating the same point. In one case a girl had contracted diphtheria after examining the clothes worn by her mother two years before when she had died of the disease. They had been kept shut up in a trunk from that time. In another instance the brush used in the throat of a diphtheritic patient was kept rolled in paper four years. It was then applied to a simple sore throat, when diphtheria soon followed. In still another instance a cradle in which a child sick with diphtheria had lain was not used for two years. It was then thought to be safe and another child was allowed to sleep in it. The result was an attack of diphtheria.

In the discussion following, Dr. A. Seibert expressed the opinion that the division into true and pseudo-diphtheritic exudations was not proper, and could not be useful clinically. Dr. J. E. Winters had observed the point mentioned by Dr. Smith regarding the development of diphtheria in scarlet fever cases, and corroborated the statement that it usually developed as the fever was declining.

In closing, Dr. Smith remarked that his reading had disclosed that wherever there was an intelligent medical profession, some preparation of iron was used in the treatment of diphtheria, generally the tincture of the chloride. In Europe the solution of the perchloride was preferred by some. The use of potassium chlorate had been discarded in France, on accout of its irritant affects, and tendency to produce nephritis. In Great Britain and America, its use had been discontinued by some, while those who still used it had reduced the dose to half a grain or a grain.

TUBERCULIN.

The title by which we are in future to know Koch's fluid is "Tuberculin." One does not hear it spoken of as frequently as formerly, and it has not yet been definitely settled what place it is to occupy in our armamentarium for the treatment of tuberculosis. I believe that the prevailing feeling here is one of disappointment. The more thoughtful, however, and those who have had long experience in the observation of tuberculosis, are inclined to give it an honorable position. Prof. Jacobi believes that it has done and can do more for tuberculosis than any other remedy except climate and surgical procedures. It has been used in a number of lupus cases at the Skin and Cancer Hospital, but no cures have yet been accomplished.

WM. L. RUSSELL.

---

Dr. W. C. Bailey, of the Post-Graduate Medical School, New York, was in Atlanta on the 12th inst., and addressed the Society of Medicine on Koch's "parataloid," or "tuberculin." Dr. Bailey spoke confidently of this new remedy, and reported several apparent cures which he had observed in New York hospitals. He emphasized the fact, however, that every case of tuberculosis is by no means adapted to this method of treatment. Patients should be operated upon only after having undergone a careful physical and clinical examination extending over several days. Dr. Bailey thought the method valuable as a means of diagnosis.

